# Key drivers of the TB epidemic in Suriname and priority actions to reduce incidence and mortality

**DOI:** 10.5588/ijtldopen.23.0535

**Published:** 2024-01-01

**Authors:** E. Commiesie, D. Stijnberg, J. van den Boogaard, F. Gopie, S. Vreden, G. de Vries

**Affiliations:** ^1^ National Tuberculosis Programme, Paramaribo,; ^2^ Anton de Kom University, Paramaribo, Suriname;; ^3^ National Institute for Public Health and the Environment (RIVM), Bilthoven, The Netherlands;; ^4^ Academic Hospital, Paramaribo, Suriname

**Keywords:** tuberculosis, epidemiology, situational analysis, HIV, national strategic plan

## Abstract

**BACKGROUND:**

The WHO has recently published updated guidance for national strategic planning for TB. To address the TB epidemic comprehensively, it is necessary to conduct an epidemiological review as part of the situation analysis in the national strategic plan.

**METHODS:**

A descriptive epidemiological study was conducted using data from the national TB register for the period of 2010–2020. Simple frequencies were calculated for demographic and clinical variables. Trends in TB notification rates for the period 2010–2020 were also calculated.

**RESULTS:**

TB notification rates between 2011 (24.3/100,000) and 2019 (23.9/100,000) remained almost the same. The HIV status was known for 97.1% of TB cases, 22.7% of whom had HIV co-infection; 10.9% of patients with detected *Mycobacterium tuberculosis* were also resistant to rifampicin. Case fatality rate for all cases was 13.0%. Of the identified contacts, 66% were screened; 28.3–47.5% of those with TB infection started treatment, 63.3–75.9% of whom completed treatment.

**CONCLUSION:**

The review identified the following areas of concern: no decline in TB rates, high proportion of TB-HIV co-infection, high rate of resistance to rifampicin, high case-fatality rates and suboptimal contact investigation care cascade . The review was used to inform interventions and key actions to reduce TB morbidity and mortality in Suriname.

## RÉSUMÉ

**CONTEXTE:**

L'OMS a récemment publié des orientations actualisées pour la planification stratégique nationale de la TB. Pour faire face à l'épidémie de TB de manière globale, il est nécessaire de procéder à un examen épidémiologique dans le cadre de l'analyse de la situation du plan stratégique national.

**MÉTHODES:**

Une étude épidémiologique descriptive a été réalisée à partir des données du registre national de la TB pour la période 2010–2020. Des fréquences simples ont été calculées pour les variables démographiques et cliniques. Les tendances des taux de notification de la TB pour la période 2010–2020 ont également été calculées.

**RÉSULTATS:**

Les taux de notification de la TB entre 2011 (24,3/100 000) et 2019 (23,9/100 000) sont restés pratiquement les mêmes. Le statut VIH était connu pour 97,1% des cas de TB, dont 22,7% présentaient une co-infection par le VIH ; 10,9% des patients chez qui *Mycobacterium tuberculosis* avait été détecté étaient également résistants à la rifampicine. Le taux de létalité pour l'ensemble des cas était de 13,0%. Parmi les contacts identifiés, 66% ont fait l'objet d'un dépistage ; 28,3–47,5% des personnes infectées par la TB ont entamé un traitement, et 63,3–75,9% d'entre elles l'ont achevé.

**CONCLUSION:**

L'étude a permis d'identifier les domaines de préoccupation suivants : absence de baisse des taux de TB, proportion élevée de co-infection TB-VIH, taux élevé de résistance à la rifampicine, taux élevé de létalité et cascade de soins sous-optimale en matière d'investigation des contacts. L'étude a été utilisée pour informer les interventions et les actions clés visant à réduire la morbidité et la mortalité dues à la TB au Suriname.

A national strategic plan (NSP) is a key document addressing the TB epidemic comprehensively through interventions within the health sector and across other sectors. The WHO recently published updated guidance for national strategic planning for TB.^1^ The document provides an outline of an NSP and describes the key considerations and steps for planning TB control activities in line with the WHO’s End TB Strategy.^2,3^ As part of the situation analysis in the NSP, it is essential to conduct an epidemiological review. This review should encompass background information on the country's TB burden and characteristics of the TB epidemic, including disease trends, mortality rates, drug resistance, relevant comorbidities, and vulnerable populations.^1^

The End TB Strategy aims to achieve a 90% reduction in global TB incidence by 2035 (compared to 2015), a 95% decrease in TB-related deaths within the same timeframe, and the elimination of catastrophic costs experienced by families affected by TB by 2020.^3^ The strategy consists of four principles, three pillars and 10 components. In addition, a set of 10 priority indicators were developed to monitor the implementation of the strategy. Suriname developed its first TB NSP for the period 2015–2020.^4^ The plan was updated in 2018 with a multisectoral and multistakeholder mid-term evaluation and was aligned with the content and indicators of the End TB Strategy.^5^ New interventions and activities were developed to reach the set targets. The NSP was revised for 2022–2026 incorporating recommendations from international reviews by the regional Green Light Committee/Pan American Health Organization (PAHO) and the latest WHO guidelines.^6^ The revised Suriname TB NSP broadly follows the content and outline of the latest WHO guidance for national strategic planning and includes a description of the country context, the national health and social care system, an epidemiological review and key domains of actions and interventions.

In brief, Suriname is a country situated on the north eastern coast of South America with a population in 2020 of 607,065 people.^7^ The country is divided into 10 districts. Almost half of the population lives in the capital district, Paramaribo, while the remaining people mostly live in the other coastal districts. The largest district is in the interior of the country and is covered by dense tropical rainforest; it comprises 80% of the country with only 7% of the population. In 2020, the HIV prevalence was 1.1% among people aged 15–49 years.^8^ According to the WHO, TB incidence was 29/100,000 population in 2020 and TB mortality 4.5/100.000.^9^ The National TB Programme (NTP) is funded by the Ministry of Health and received additional support from the Global Fund from 2010 until 2021.

The aim of the present study is to describe the factors that are key drivers of the TB epidemic in Suriname and discuss priority domains of action and interventions to reduce TB incidence and mortality in the country.

## METHODS

### Data sources

This was a descriptive epidemiological study using data from the national TB register, which is an electronic database (MySQL) maintained at the NTP office. The data include the demographic and clinical variables of cases notified between 2010 and 2020. The following data were collected from the national TB register: year of registration, sex, age category, TB case type, site of TB disease, whether or not TB was bacteriologically confirmed, rifampicin (RIF) drug susceptibility test results, clinical risk factors (HIV and diabetes mellitus [DM]), structural risk factors (drug or alcohol abuse) and treatment outcome. Information on the management of people with TB infection was available for 2019 and 2020.

### Settings

People with presumptive TB are referred by a family doctor to either the pulmonologist or the NTP for TB diagnosis but can also visit the NTP without referral. In remote areas, sputum is collected and transported to the central laboratory for examination. Xpert^®^ MTB/RIF (Cepheid, Sunnyvale, CA, USA) testing was introduced in Suriname in April 2012. Since then, the diagnostic algorithm has included the examination of two specimens using smear microscopy, culture and Xpert. This algorithm was updated in 2021 with Xpert as the only initial diagnostic test to evaluate people with presumptive TB, in line with international recommendations.^10,11^ TB treatment is initiated by the pulmonologist according to national guidelines based on international standards for persons-centred care,^12^ while the NTP provides treatment follow-up after discharge of the patient, preferably under supervision (directly observed treatment/video-observed treatment).

All close contacts of sputum smear-positive TB patients are eligible for contact investigation in Suriname. Non-close contacts who are eligible include children aged <5 years, contacts with symptoms and contacts with immune suppression. Contacts are screened using a tuberculin skin test (TST) after the TB diagnosis of the index patient, and again, 8–12 weeks later if the initial TST is negative. Contacts with TST ≥10 mm (≥5 mm in children <5 years of age and in people living with HIV [PLHIV]) are referred to a pulmonologist or paediatrician to exclude TB disease and start TB preventive treatment (TPT). Data on TB contact investigations were available for the period covering 2016 to 2020, initially documented on paper and then electronic since 2018. Since 2019, a paper-based TPT register has been maintained for people starting TPT. The TB contact investigation data were merged with the data from the TPT register.

### Data analysis and statistics

WHO guidelines on definitions were used in this study.^12^ A patient was classified as a new case if the person had not been treated for TB before or did not receive anti-TB drugs for more than 1 month before the current diagnosis. All other patients were classified as ‘previously treated’, which included relapse patients (patients previously completing TB treatment) and patients starting treatment after loss to follow-up. A bacteriologically confirmed case was defined as a case with a specimen positive on smear microscopy, culture or Xpert.^13^

Simple frequencies were calculated for demographic and clinical characteristics of patients notified in 2015–2020, and for the two reference years (2015 and 2020) to monitor the progress of TB control. The estimated TB incidence rates were taken from the accompanying CSV files published with the 2022 WHO Global Tuberculosis Report (https://www.who.int/teams/global-tuberculosis-programme/data).^9^ The annual notification rates for the period 2010–2020 were calculated by dividing the number of notified TB cases and the estimated population in Suriname according to the Population Division of the United Nations.^7,9^

### Ethical approval

The publication of these results was approved by the Suriname Director of Health, Paramaribo, Suriname, the main authority in the country for approving research with human subjects.

## RESULTS

**Figure 1.**
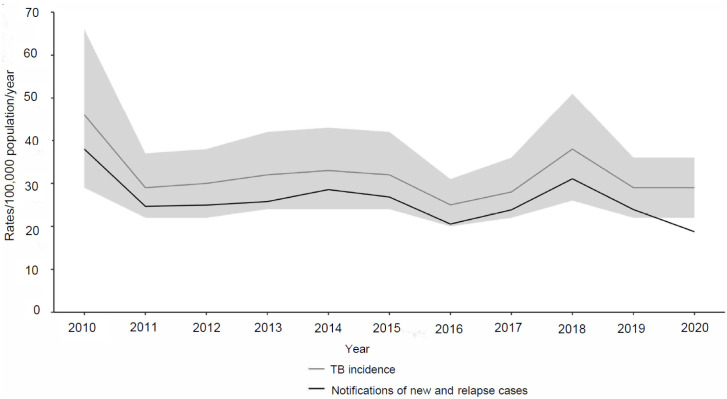
Trends in estimated TB incidence and notification rates in Suriname, 2010–2020. Shaded area represents 95% uncertainty intervals of the WHO-estimated TB incidence rate. Source: WHO country data profile: Suriname.^9^

The number of notified TB cases varied from 201 in 2010 to 150 in 2015 and 110 in 2020, corresponding to notification rates of respectively 36.8, 26.1 and 18.1/100,000 in 2010, 2015 and 2020 (Figure 1). The notification rate remained almost the same between 2011 (24.3/100,000) and 2019 (23,9/100,000). The reported numbers in 2010 and 2020 were outliers. The WHO estimated that 83% of annual TB cases in Suriname are detected; the TB incidence thus precisely mirrors the trend in the TB notification rate (Figure 1). For 2020, the WHO maintained the incidence rate of 2019, despite a decrease in the number of notifications, suggesting that only 65% of TB cases were notified in the first year of the COVID-19 pandemic.

**Figure 2.**
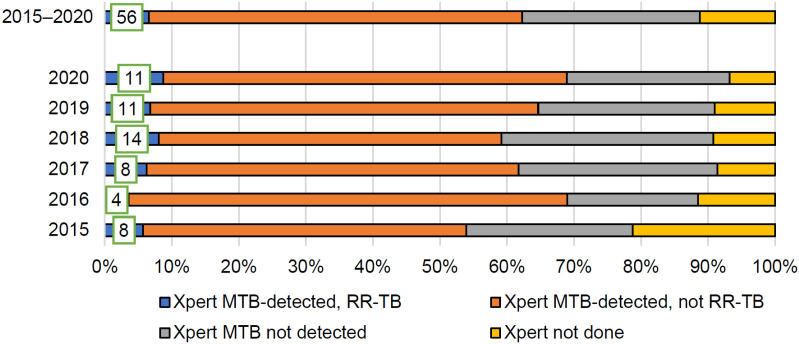
Proportion of cases tested with WHO-recommended rapid tests (Xpert MTB/RIF) and proportion/cases with positive results, including rifampicin resistance in Suriname, 2015–2020. MTB = *Mycobacterium tuberculosis*; RR-TB = rifampicin-resistant TB.

In total, 830 patients were notified in the period 2015–2020 (Table 1). Most of the patients were male (70.8%), aged between 25 and 64 years (71.8%), and almost all were born in Suriname (95.1%). Most patients (84.7%) had pulmonary TB; 89 (10.7%) had previously been treated for TB; 730 (88.0%) were examined using at least one Xpert test. In 512 patients, *Mycobacterium tuberculosis* was detected, and 56 of these (10.9%) had RIF resistance (Figure 2). In addition, another 75 had either a positive culture for *M. tuberculosis* or positive acid-fast bacilli on auramine test result. Thus, 587/830 (70.7%) patients were bacteriologically confirmed.

**Table 1.** Characteristics of TB patients in Suriname, 2015–2020.

	2015-2020	2015	2020
	(*N* = 830)	(*N* = 150)	(*N* = 110)
	*n* (%)	*n* (%)	*n* (%)
Sex			
Male	588 (70.8)	101 (67.3)	77 (70.0)
Female	242 (29.2)	49 (32.7)	33 (30.0)
Age groups, years			
<5	35 (4.2)	3 (2.0)	4 (3.6)
5-14	29 (3.5)	6 (4.0)	1 (0.9)
15-24	97 (11.7)	10 (6.7)	10 (6.1)
25-44	279 (33.6)	55 (36.7)	46 (41.8)
45-64	317 (38.2)	58 (38.7)	44 (40.0)
≥65	73 (8.8)	18 (12.0)	5 (4.5)
Country of birth			
Suriname	789 (95.1)	139 (92.7)	103 (93.6)
Other	39 (4.7)	10 (6.7)	7 (6.4)
Unknown	2 (0.2)	1 (0.7)	
Site of disease			
Pulmonary	703 (84.7)	123 (82.0)	96 (87.3)
Extrapulmonary	127 (15.3)	27 (18.0)	14 (12.7
Case type			
New	741 (89.3)	131 (87.3)	93 (84.5)
Previously treated	89 (10.7)	19 (12.7)	17 (15.5)
Bacteriologically confirmed			
No	243 (29.3)	50 (33.3)	26 (23.6)
Yes	587 (70.7)	100 (66.7)	84 (76.4)
Rifampicin drug susceptibility			
Susceptible	453 (54.6)	70 (46.7)	65 (59.1)
Resistant	56 (6.7)	8 (5.3)	11 (10.1)
Unknown	321 (38.7)	72 (48.0)	34 (30.9)
HIV status			
Negative	623 (75.1)	105 (70.0)	82 (74.6)
Positive	183 (22.0)	43 (28.0)	25 (22.7)
Unknown	24 (2.9)	3 (2.0)	3 (2.7)
Diabetes mellitus			
No or unknown	708 (85.3)	134 (89.4)	94 (85.5)
Yes	122 (14.7)	16 (10.6)	16 (14.5)
Substance abuse			
No or unknown	477 (57.5)	85 (56.7)	65 (59.1)
Alcohol, drugs or both	353 (42.5)	65 (43.3)	45 (40.9)

**Figure 3.**
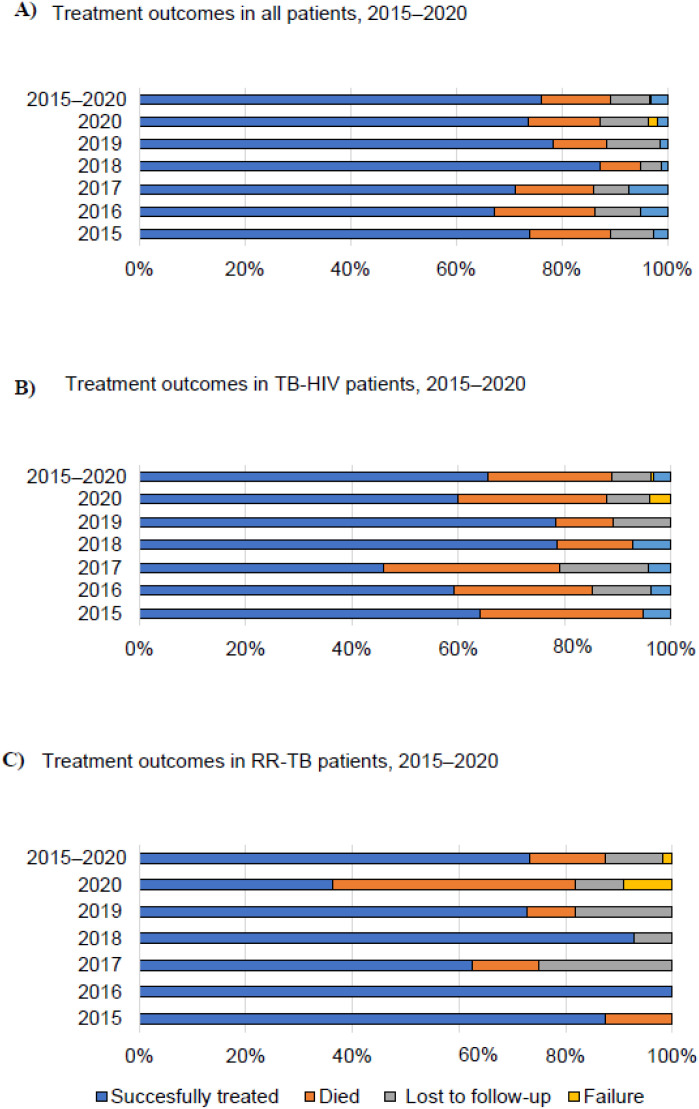
Treatment outcomes in **A)** all TB patients, **B)** TB-HIV patients, and **C)** RR-TB patients in Suriname, 2015–2020. The successfully treated is the sum of cured and completed. RR-TB = rifampicin-resistant TB.

In 2015–2020, 97.1% of TB cases had known HIV status. Of those tested, 22.7% had an HIV co-infection. The proportion of TB-HIV patients on antiretroviral therapy (ART) in 2015–2020 was 73%. The ART coverage of TB-HIV patients increased from 63% in 2016 to 89% in 2019, but this proportion dropped to 72% in 2020. Information on screening PLHIV for TB disease and TB infection was not systematically recorded. Table 1 also provides information on other comorbidities: 14.7% of patients had DM and 42.5% had substance abuse. Overall, 76% of all TB patients were successfully treated during 2015–2020 (Figure 3A). Figure 3B and 3C show the treatment outcome results for respectively TB-HIV patients (64% successful outcome) and RR-TB patients (73% successful outcome). Case fatality was 13.0% for all patients, 14.3% for RR-TB patients and 23.0% for TB-HIV patients. In 2016–2020, 66% of identified contacts were screened for TB and TB infection (2–3 contacts per TB patient) (Table 2). Of these contacts, 1.4% had TB and 36.6% had TB infection. Respectively 28.3% and 47.5% of eligible contacts started TPT in 2019 and 2020, while 63.3% and 75.9% completed TPT for the same years.

**Table 2.** Cascade of care in TB contact investigations in Suriname, 2016–2020.

	2016	2017	2018	2019	2020	Total
	*n* (%)	*n* (%)	*n* (%)	*n* (%)	*n* (%)	*n*/*N* (%)
TB patients, *n*	116	136	179	139	110	680
Contacts identified,*n*	566	1,053	499	589	366	3,073
Contacts screened	280 (49.5)	831 (78.9)	403 (80.8)	345 (58.6)	163 (44.5)	2,022 (65.8)
TB cases identified	0 (0)	11 (1.3)	13 (3.2)	3 (0.8)	2 (1.2)	29 (1.4)
TB infections (TST ≥10 mm) identified	111 (39.6)	253 (30.4)	209 (51.9)	106 (30.7)	61 (37.4)	740 (36.6)
Persons with TB infection starting TPT	Unknown	Unknown	Unknown	30 (28.3)	29 (47.5)	59/165 (35.3)
Persons completing TPT	Unknown	Unknown	Unknown	19 (63.3)	22 (75.9)	41/59 (69.5)

TPT = TB preventive treatment; TST = tuberculin skin test.

## DISCUSSION

TB rates are not decreasing in Suriname. Our review highlights a number of characteristics of TB patients which are of concern, such as the high proportion of TB patients co-infected with HIV (23%), the high proportion of patients with structural risk factors (43%), the high rate of RIF resistance (10%), a high case fatality rate (13%) and suboptimal contact investigation. Our analysis of the epidemiological situation guided the implementation of interventions and critical measures to reduce TB incidence and mortality. We have recently outlined the challenges and opportunities in Suriname's journey towards TB elimination, emphasising the significance of continuous review and strategic planning.^14^

The main reason why TB incidence has not declined in the country is ongoing transmission, given that almost all cases are born in Suriname and are of relatively young age. The high proportion of pulmonary TB patients with positive acid-fast bacilli smears and the high case fatality rate suggest that people with TB are diagnosed late. No data are, however, collected on patient and diagnostic delay factors, such as duration of symptoms. The proposed key actions in the Suriname TB NSP include TB awareness raising campaigns involving community-based organisations to educate the community, improving access to healthcare services for people with presumptive TB, training healthcare providers in TB symptom recognition and the use of WHO-recommended rapid tests in people with presumptive TB.

One key intervention of the End TB Strategy is systematic screening of contacts.^3,15^ Our cascade of care analysis shows that substantial proportions of contacts are lost at every step of contact investigation: one-third of the identified contacts were not screened, and only 28% of people with TB infection started TPT in 2019, and 48% in 2020. TB disease and TB infection rates observed in contacts in our study (1.4% and 37%, respectively) were lower than those reported in two systematic reviews in low- and middle-income countries, which reported rates of TB disease of respectively 3.3% and 2.9%, and of respectively 51.5% and 43.8% for TB infection.^16,17^ Both systematic reviews, as well as our study, demonstrate the high value of contact investigation in TB control. Two interventions were thus included in the revised NSP to improve the effectiveness of contact investigation; both were implemented in October 2022. First, a person-centred ‘one-stop approach’ was introduced with contacts now receiving medication immediately after reading the (positive) TST and after exclusion of disease. Second, national guidelines on TPT were updated according to the latest WHO guidelines now recommending shorter rifamycin-containing regimens.^18^ A more proactive approach to reach contacts identified is recommended in the NSP with reminders, incentives or enablers to patients in need.

The epidemiological review also identified a number of high-risk groups for TB such as PLHIV and people with substance abuse. DM has been described as a risk factor for the development of TB; however, in our study DM prevalence among TB patients (15%) was similar to the prevalence in the general population.^19,20^ The proportion of TB patients co-infected with HIV in Suriname (23%) is much higher than the regional (11%) or global average (8%).^21^ Although ART alone can reduce the risk of TB disease by 65%, PLHIV remain substantially at higher risk for TB even when they are on ART and with high CD4 cell counts.^22^ TPT has a synergistic effect with ART and also independently lowers the risk of TB disease among PLHIV.^22–24^ In line with WHO guidance, the NSP recommends, among others, screening PLHIV for TB disease at every health visit, early initiation of ART in TB patients diagnosed with HIV, testing PLHIV for TB infection and providing TPT to those infected.^19,25,26^

Other key drivers, such as alcohol and drug use, are also both highly prevalent among TB patients in Suriname. It is well-known that TB is a disease affecting the poor and socially excluded people.^27^ The NSP advises the development of screening strategies to reach these populations in collaboration with stakeholders and involving the key populations, including a monitoring and evaluation system to assess their effectiveness. The NSP also supports efforts for poverty reduction and the resolution of fundamental social determinants contributing to TB, which is not easy, as the country is currently facing economic hardship.

A high proportion of patients with known drug susceptibility status in our study had RIF resistance. In 2021, the reported RIF resistance rate was 11% in Suriname and the highest in the region of the Americas.^9^ Gopie et al. analysed in their study that all had low-level RIF resistance caused by D435Y *rpo*B mutation and all but one had isoniazid susceptibility.^28^ Their proposed intervention, i.e., standard treatment with high-dose RIF, is currently implemented under research conditions. Treatment is initiated by a dedicated pulmonologist and discussed in a Concilium of national and international TB experts.

Our epidemiological review also indicates high TB case fatality rates (10%), similar for patients with drug-susceptible and drug-resistant TB, and very high rates for HIV-coinfected TB patients (23%). Stijnberg et al. analysed the factors associated with mortality in TB-HIV patients and found that patients on ART or on supervised TB treatment had lower mortality.^29^ Their study reported a median of nearly 1.5 years between the time of HIV diagnosis and TB diagnosis for 80% of the TB-HIV patients, which suggests that opportunities for preventing TB using TPT exist. Apart from reinforcing the TB-HIV collaborative activities, the NSP advises to organise regular meetings to discuss fatal cases and identify options to prevent these.

### Strengths and limitations

The strength of our study is that a comprehensive set of national TB data was used to identify gaps in TB control in Suriname. Some data, however, were incomplete (e.g., data on contact investigation) or lacking (e.g., data on the CD4 count or viral load in TB-HIV patients). While an epidemiological review is tailored to the specifics of each country and its utilisation in a TB NSP, the principles of such a review are broadly applicable.^30^ Consequently, our study can be viewed as an illustration of the national adaptation of the End TB Strategy.

## CONCLUSION

This study shows that a review of the TB epidemiological situation with WHO guidance on national strategic planning helps in recognising the challenges faced by NTPs and facilitates the formulation of strategies to tackle these challenges.
